# Association between the triglyceride-glycated hemoglobin index and diabetes risk among patients with Non-alcoholic fatty liver disease: A longitudinal cohort study

**DOI:** 10.1371/journal.pone.0350633

**Published:** 2026-06-05

**Authors:** Cuimei Wei, Changchun Cao, Zengxin Xu, Gang Chen, Wenjing Liu, Miao Zhang, Haofei Hu, Shanghua Xu

**Affiliations:** 1 Department of Cardiology, Nanping First Hospital Affiliated to Fujian Medical University Nanping Fujian Province, China; 2 Department of Geriatrics, Shenzhen Second People’s Hospital, Shenzhen, Guangdong Province, China; 3 Department of Rehabilitation, Shenzhen Second People’s Hospital, Shenzhen Second People’s Hospital Dapeng Hospital, Shenzhen, Guangdong Province, China; 4 Bagualing Community Health Service Center, Shenzhen Futian District Second People’s Hospital, Shenzhen, Guangdong Province, China; 5 Department of Nephrology, Shenzhen Second People’s Hospital, The First Affiliated Hospital of Shenzhen University, Shenzhen, Guangdong Province, China; University of Montenegro-Faculty of Medicine, MONTENEGRO

## Abstract

**Background:**

Emerging evidence suggests that the triglyceride-glycated hemoglobin index (TyH-i) may be a novel predictor of type 2 diabetes (T2D) risk. However, in patients diagnosed with NAFLD, the precise link between TyH-i and T2D is still not well elucidated. Currently, the literature lacks sufficient research on how this index affects the incidence of diabetes in the NAFLD population, highlighting a significant gap in our understanding of the interactions between these metabolic conditions. This research seeks to evaluate the association between the TyH-i and diabetes risk, as well as to investigate its predictive capability in patients with NAFLD concerning the triglyceride-glucose index (TyG-i).

**Methods:**

Based on the data of 2741 participants who had no diabetes at baseline and suffered from NAFLD, leveraging a nationwide retrospective cohort, we derived the TyH-i as the natural logarithm of [HbA1c (%) × fasting TG (mg/dL) ÷ 2].To assess the relationship between TyH-i and the risk of developing T2D, a Cox proportional hazards regression model was used to estimate the hazard ratio (HR) and 95% confidence interval (CI). Furthermore, the nonlinear correlations between them were also investigated using the restricted cubic spline model. We gauged the relative discriminatory capacity of TyH-i versus TyG-i by constructing time-dependent receiver-operating characteristic (ROC) plots and comparing the corresponding areas under the curve (AUC) with their 95% CI.

**Results:**

In a median follow-up duration of 5.21 years, incident diabetes was recorded in 223 individuals. The multivariable-adjusted analysis revealed that each unit increase in TyH-i corresponds to a 46% elevation in diabetes risk (HR: 1.75; 95%CI: 1.28-2.41; P = 0.0005). Additionally, a U-shaped relationship was identified between TyH-i levels and the occurrence of type 2 diabetes. Specifically, TyH-i values below 4.95 displayed a notable inverse association with the risk of type 2 diabetes (HR: 0.20, 95%CI: 0.05-0.78, P = 0.0206). Conversely, an elevation in TyH-i levels beyond this threshold was linked to a higher risk of developing type 2 diabetes (HR: 2.01, 95% CI: 1.45-2.39, P < 0.0001). Furthermore, the findings indicate that the Youden Index for both TyH-i and TyG-i is nearly identical, implying that TyH-i may serve as a valid marker for diabetes diagnosis.

**Conclusion:**

Among the patient population suffering from NAFLD, the initial TyH-i level demonstrated a U-shaped correlation with the emergence of type 2 diabetes. This indicated inflection point can act as a practical clinical threshold, allowing differentiation between individuals at low and high risk. The findings imply that keeping TyH-i close to this inflection may play a role in mitigating the progression to diabetes among NAFLD patients.

## Introduction

Type 2 diabetes（T2D） is a chronic metabolic disorder defined by persistent elevation of blood glucose; it has rapidly evolved into a worldwide public-health crisis. Globally, the frequency of diabetes mellitus continues to climb among Chinese adults; recent surveys indicate that 12.8% of the population is affected [1]. Research predictions indicate that by the year 2045, the worldwide number of individuals living with diabetes mellitus will exceed seven hundred million [2]. Diabetes mellitus markedly amplifies the probability of a spectrum of complications, encompassing elevated mortality, cardiovascular disorders, sight-threatening retinopathy, unfavorable stroke prognosis, and progressive chronic kidney disease [3–5].

Non-alcoholic fatty liver disease(NAFLD) is an inclusive designation that spans the entire spectrum, beginning with isolated hepatic lipid accumulation-termed metabolic dysfunction–associated steatosis and extending to the inflammatory phenotype known as metabolic dysfunction–associated steatohepatitis. Recent studies reveal a complex, bidirectional interplay between T2D and NAFLD [6], and T2D functions as a pivotal driver of NAFLD [7], with roughly one in two individuals with T2D presenting histological or imaging evidence of hepatic steatosis [8]. Additionally, the presence of NAFLD itself amplifies the likelihood of incident T2D, underscoring a reciprocal pathogenic loop between these two metabolic disorders [9]. Recent research indicates that individuals affected by NAFLD face an increased susceptibility to developing diabetes compared to the general population [10]. Consequently, it is essential to comprehend the underlying risk elements contributing to glucose dysregulation in NAFLD patients. This understanding can inform the creation of effective strategies to avert the onset of diabetes.

Over the past decade, composite metabolic scores that simultaneously capture dyslipidaemia and glycaemic dysregulation have emerged as pragmatic instruments for estimating future diabetes risk. Foremost among these, the triglyceride-glucose index (TyG-i) stands out as the most thoroughly validated and is widely regarded as a reliable surrogate indicator of insulin resistance as well as a predictor of new-onset type 2 diabetes mellitus [11–15]. Nonetheless, a key shortcoming of the TyG-i is its disregard for sustained glycaemic regulation determinant integral to the natural history of diabetes-thereby constraining its capacity to reflect long-term metabolic trajectory. Unlike isolated fasting glucose values, glycated hemoglobin (HbA1c) integrates prevailing glycaemia across the preceding eight to twelve weeks and therefore furnishes a more holistic appraisal of long-term glucose homeostasis [16]. Dyslipidemia and abnormal glucose metabolism are core pathological drivers of metabolic disorders, systemic inflammation, and atherosclerotic progression. Triglycerides (TG) are a key marker of lipid metabolism disturbance, reflecting circulating lipid accumulation and insulin resistance at the peripheral level. HbA1c mirrors long-term average blood glucose levels, chronic glycemic toxicity, and sustained pancreatic β-cell dysfunction, which can independently aggravate endothelial injury and metabolic deterioration. Given the close interaction and synergistic pathogenic effects between lipid and glucose metabolism disorders, a single lipid or glucose indicator cannot fully reflect the holistic metabolic status of individuals. The triglyceride and glycated hemoglobin index (TyH-i), as a novel composite metabolic indicator, integrates peripheral lipid overload reflected by triglycerides and long-term glycemic dysregulation represented by HbA1c. The biological rationale for combining these two biomarkers lies in their shared upstream mechanism of insulin resistance and complementary predictive values: co-elevated TG and HbA1c jointly exacerbate oxidative stress, chronic low-grade inflammation and vascular endothelial dysfunction, and the combined index can more comprehensively capture integrated metabolic abnormalities than single indicators. Although HbA1c has been widely recognized as an important indicator for assessing blood sugar levels, studies that incorporate it into comprehensive metabolic scoring systems aimed at predicting the onset of diabetes have received relatively little empirical attention. A recent large-sample longitudinal study has shown that an increase in the level of TyH-i raises the risk of diabetes and is slightly better than TyG-i in predicting diabetes [17]. Despite growing interest, the relationship between the TyH-i and type 2 diabetes risk among individuals with NAFLD has been scarcely investigated. To address this gap, we conducted a retrospective cohort analysis to evaluate the longitudinal association between TyH-i and the development of T2D in patients diagnosed with NAFLD.

## Materials and methods

### Ethics approval and consent to participate

We analyzed longitudinal data from the NAGALA cohort, comprising participants prospectively enrolled at Murakami Memorial Hospital (Gifu, Japan) from May 1st, 1994 to December 31st, 2016. De-identified datasets were obtained from the Dryad Digital Repository and handled in strict accordance with its data-use policy. The original study has been approved by the institutional ethics review committee of Murakami Memorial Hospital, and all participants provided written informed consent [18]. This second analysis does not require separate approval from the institutional ethics committee; the entire research process strictly adheres to the principles of the Helsinki Declaration and complies with current norms and regulatory requirements.

### Data source and Study participants

**Fig 1:**
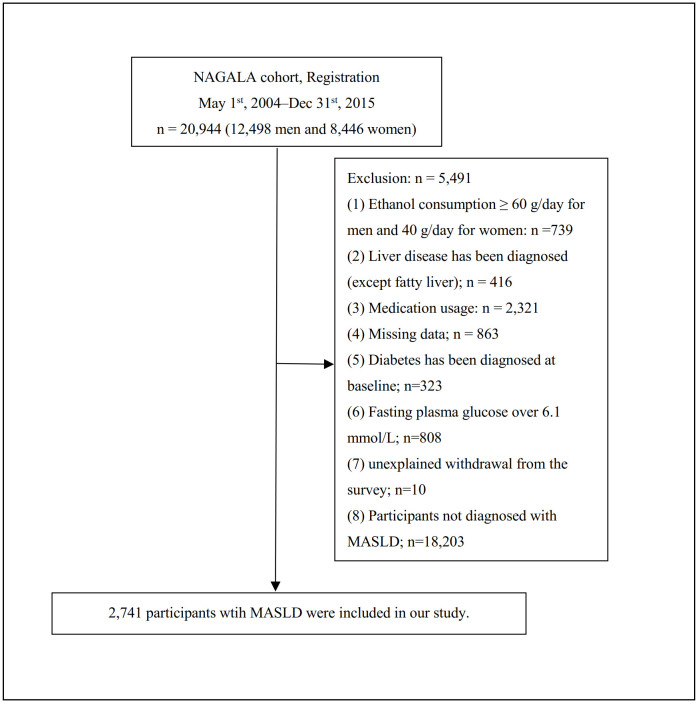
Flowchart of study participants.

The study population was restricted to individuals who completed a minimum of two consecutive health examinations at Murakami Memorial Hospital [18]. According to the exclusion criteria of the original study, participants meeting any of the following conditions will be excluded: those currently taking of any medication, pre-existing liver disease, missing data, excessive alcohol consumption at baseline, missing fasting plasma glucose (FPG) values, a confirmed diagnosis of diabetes at baseline, not diagnosed with NAFLD, or unexplained withdrawal from the study. Initially, a total of 20,944 people were included. After applying the exclusion criteria, a total of 2,741 individuals were finally included in our study (Fig 1).

### Data collection

The baseline data were collected during routine health check-ups by trained staff, including demographic, clinical and laboratory variables. The resulting dataset encompassed demographic attributes (age, sex), anthropometrics (body mass index (BMI), height, weight), lifestyle factors (cigarette use, alcohol consumption, leisure-time physical activity), pressure value (systolic and diastolic arterial pressure), and an extensive biochemical profile that comprised FPG, total cholesterol(TC), alanine aminotransferase(ALT)，high-density lipoprotein cholesterol(HDL-C) and aspartate aminotransferase (AST).Standardised self-administered questionnaires [18] captured sociodemographic characteristics, and all biochemical indices were quantified on a fully automated analyser to guarantee precision and uniformity across the cohort. The TyH-i was calculated as follows: the natural logarithm of the product of HbA1c (percent) and fasting triglycerides (mg/dL), with the product divided by 2 before log transformation: TyH-i = ln[HbA1c × TG/2]. The TyG-i was analogously calculated by substituting glycated hemoglobin with FPG (mg/dL): TyG-i = ln[(FPG × TG / 2] [13].

### Outcome measures

Diabetes was ascertained based on any of the following: a self-reported history of physician-diagnosed diabetes, glycated hemoglobin (HbA1c) ≥ 6.5%, or fasting plasma glucose (FPG) ≥ 7.0 mmol/L[19].

### Statistical analysis

We summarized baseline characteristics with standard descriptive statistics. Continuous variables are presented as either mean ± SD or median (inter-quartile range), depending on distribution symmetry; categorical variables are expressed as counts and proportions. Group comparisons for categorical factors employed the χ² test, whereas continuous measures were evaluated with one-way ANOVA or the Kruskal–Wallis H statistic, as dictated by normality diagnostics.

Following STROBE recommendations, we built a trio of nested Cox proportional-hazards models to quantify the association between TyH-i and incident diabetes. Model 1 was crude, not adjusting for any variables. Model 2 added standard demographic and lifestyle covariates: age, sex, smoking, exercise habits, systolic blood pressure (SBP), and alcohol consumption. Model 3 further adjusted for BMI, ALT, AST, gamma-glutamyl transpeptidase(GGT), HDL-C, TC, and FPG. As show In S1 Table, collinearity diagnostics revealed that diastolic blood pressure (DBP) was highly correlated with other predictors, so it was omitted from the final specification.

To test result stability, we conducted sensitivity checks that sequentially excluded participants who either drank or whose BMI reached or exceeded 30 kg/m², thereby accounting for the established heightened diabetes risk conferred by alcohol consumption and excess adiposity [19].

To assess potential nonlinearity in the relationship between TyH-i and diabetes incidence, we first applied penalized splines for smooth curve estimation and incorporated restricted cubic splines within the Cox proportional hazards framework. When evidence of a nonlinear pattern emerged, inflection points were identified via a recursive search procedure, and segmented (two‑piecewise) Cox models were fitted on either side of the identified thresholds. The best model characterizing the association between TyH-i and diabetes was determined through the log-likelihood ratio test.

Further stratified analyses were stratified by alcohol consumption, sex, age, body mass index, exercise habit, and smoking status; interaction effects across these subgroups were examined through likelihood-ratio testing.

The comparative discriminative performance of TyH-i versus TyG-i was evaluated using receiver operating characteristic (ROC) analysis, with the area under the curve (AUC) as the primary summary measure. Reporting conformed to STROBE guidance. All computations were performed in EmpowerStats, and statistical significance was defined as a two-sided P < 0.05.

## Results

### Participant characteristics

The analytic cohort comprised 2,741 adults free of diabetes at enrolment (mean age 44.8 ± 8.29 years; 82.3% male). As shown in Table 1, participants were distributed into TyH-i quartiles. The uppermost quartile (Q4) was characterised by markedly elevated aminotransferases (ALT, AST, GGT), fasting lipids (TG and TC), indices of chronic glycaemia (HbA1c, FPG), blood pressure (SBP, DBP), BMI, and habitual alcohol consumption. Moreover, this quartile displayed diminished HDL-C, a greater prevalence of smokers, and a higher proportion of males.

**Table 1:** The baseline characteristics of participants.

TYH Quartile	Q1(3.60-5.32)	Q2(5.32-5.68)	Q3(5.69-6.04)	Q4(6.04-7.59）	P-value
**Participants**	685	685	685	686	
**Age(year)**	44.66 ± 8.63	45.26 ± 8.40	44.85 ± 8.13	44.45 ± 8.00	0.315
**BMI(kg/m**^**2**^)	24.78 ± 2.95	25.42 ± 3.42	25.71 ± 3.06	26.03 ± 2.81	<0.001
**SBP(mmHg)**	121.24 ± 14.17	122.88 ± 14.57	124.30 ± 14.62	126.67 ± 15.25	<0.001
**DBP(mmHg)**	76.09 ± 9.98	77.43 ± 9.91	78.56 ± 9.98	80.43 ± 10.36	<0.001
**ALT**	24.00(18.00-33.00)	26.00(19.00-36.00)	28.00(21.00-40.00)	32.00(23.00-45.00)	<0.001
**AST**	20.98 ± 9.29	21.63 ± 9.61	22.95 ± 10.43	24.60 ± 10.02	<0.001
**GGT**	19.00(14.00-26.00)	22.00(16.00-32.00)	24.00(18.00-36.00)	29.00(21.00-43.00)	<0.001
**HbA1c(%)**	5.24 ± 0.32	5.29 ± 0.33	5.30 ± 0.34	5.35 ± 0.34	<0.001
**FPG(mg/mL)**	5.34 ± 0.37	5.38 ± 0.36	5.41 ± 0.36	5.48 ± 0.34	<0.001
**TG(mg/mL)**	0.69 (0.55-0.79)	1.06 (0.96-1.15)	1.49 (1.38-1.63)	2.33 (2.02-2.87)	<0.001
**TC(mg/mL)**	5.05 ± 0.82	5.35 ± 0.76	5.60 ± 0.85	5.79 ± 0.85	<0.001
**HDL-c(mg/mL)**	1.36 ± 0.34	1.22 ± 0.27	1.14 ± 0.23	1.03 ± 0.22	<0.001
**follow-up time(years)**	5.11 (2.36-9.20)	5.02 (2.41-9.64)	5.84 (2.61-9.94)	5.08 (2.05-9.21)	0.686
**Total alcohol consumption**	1.00 (0.00-44.00)	1.00 (0.00-60.00)	4.20 (1.00-79.00)	(1.00-84.00)	<0.001
**Gender**					<0.001
**Female**	171 (24.96%)	153 (22.34%)	90 (13.14%)	72 (10.50%)	
**Male**	514 (75.04%)	532 (77.66%)	595 (86.86%)	614 (89.50%)	
**Smoking**					<0.001
**never**	361 (52.70%)	330 (48.18%)	281 (41.02%)	254 (37.03%)	
**past**	171 (24.96%)	198 (28.91%)	183 (26.72%)	174 (25.36%)	
**current**	153 (22.34%)	157 (22.92%)	221 (32.26%)	258 (37.61%)	
**Incident of DM (%)**	31 (4.53%)	45 (6.57%)	57 (8.32%)	90 (13.12%)	<0.001
**Habit of exercise(%)**	112 (16.35%)	110 (16.06%)	95 (13.87%)	84 (12.24%)	0.103

Values are n (%) or mean ± SD

Abbreviations. BMI, body mass index;; HDL-c, high-density lipoprotein cholesterol; FPG, fasting plasma glucose; TC, total cholesterol; TG, triglyceride; SBP, systolic blood pressure;DBP, diastolic blood pressure; HbA1c, glycated hemoglobinc;ALT, alanine aminotransferase;AST,aspartate aminotransferase;GGT,gamma-glutamyl transpeptidase

### The incidence rate of DM

Over a median follow-up of 5.21 years, 223 new diabetes cases were identified, corresponding to a cumulative incidence of 8.14% (95% CI: 7.11-9.16) and an incidence rate of 13.56 per 1,000 person‑years (Table 2). Both cumulative risk and incidence rate increased progressively across TyH‑i quartiles. Participants in the highest TyH‑i category (Q4) experienced the greatest burden, with a cumulative incidence of 13.12% (95% CI: 10.59-15.65) and an event rate of 22.05 per 1,000 person‑years. In contrast, those in the lowest quartile (Q1) exhibited the smallest values 4.53% (95% CI: 2.97-6.09) and 7.62 per 1,000 person‑years, respectively. A statistically significant positive trend was evident across increasing TyH‑i quartiles.

**Table 2:** The incidence rate of diabetes.

TyH-i	Participants(n)	Diabetes events(n)	Cumulative incidence (95% CI)(%)	Per 1,000 person-year
Total	2741	223	8.14 (7.11-9.16)	13.56
Q1	685	31	4.53 (2.97-6.09)	7.62
Q2	685	45	6.57 (4.71-8.43)	11.00
Q3	685	57	8.32 (6.25-10.39)	13.53
Q4	686	90	13.12 (10.59-15.65)	22.05
P for trend				<0.01

TyH-i: triglyceride-glycated hemoglobin index; CI: confidence interval

### Kaplan-Meier survival curve

**Fig 2:**
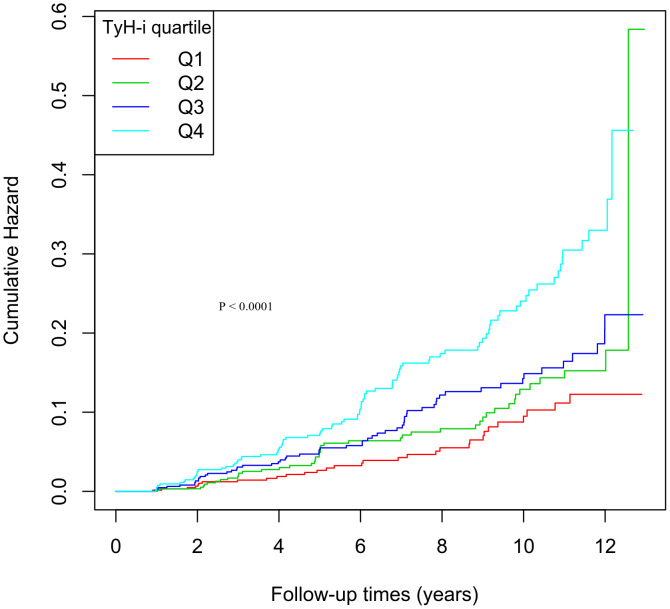
Kaplan-Meier survival curve.

Kaplan-Meier plots presented in Fig 2 delineate the stepwise elevation in diabetes risk across TyH-i quartiles. The analysis results show that as the quartiles increase, the risk of diabetes exhibits a clear gradient change: the higher the TyH-i level, the significantly greater the cumulative risk of diabetes (log-rank P < 0.0001). Subjects in the top quartile exhibited the sharpest rise in incident events over follow-up, whereas the bottom quartile remained on the lowest trajectory throughout the observation window.

### The results of the effect of TyH-i levels on diabetes

Table 3 summarises the association between TyH-i values and incident diabetes across three analytical models. S2 Table shows the relationship between TG and HbA1C as independent predictors and the incidence of diabetes. In the unadjusted model, the increase in TyH-i is associated with an increased risk of diabetes. (HR: 2.08; 95% CI: 1.62-2.67; P < 0.0001). After adjustment in Model 2 for age, sex, cigarette use, alcohol consumption, exercise habits, BMI, and SBP, the relationship remained materially unchanged (HR: 1.97; 95%CI: 1.51-2.56; P < 0.0001). In the fully adjusted specification (Model 3), which additionally controlled for hepatic enzymes (ALT, GGT, AST), and lipid measures (HDL-C, TC), TyH-i remained independently associated with new-onset diabetes (HR = 1.75; 95% CI: 1.28-2.41; P = 0.0005). Furthermore, relative to the lowest TyH-i quartile (Q1), participants in the top quartile (Q4) had a 1.06-fold higher risk of developing diabetes (HR = 2.06; 95% CI: 1.27-3.34, P for trend = 0.0014).

**Table 3:** The relationship between TyH-i and the onset of diabetes under different models.

Variable	Model 1 HR 95%CI P	Model 2 HR 95%CI P	Model 3 HR 95%CI P
diabetes			
TyH-i	2.08 (1.62, 2.67) < 0.0001	1.97 (1.51, 2.56) < 0.0001	1.75 (1.28, 2.41) 0.0005
**TyH-i:(Quartile)**			
**Q1**	Ref.	Ref.	Ref.
**Q2**	1.42 (0.90, 2.24) 0.1361	1.32 (0.83, 2.09) 0.2367	1.24 (0.78, 1.99) 0.3667
**Q3**	1.73 (1.12, 2.68) 0.0140	1.59 (1.02, 2.47) 0.0396	1.42 (0.88, 2.29) 0.1521
**Q4**	2.88 (1.91, 4.33) < 0.0001	2.51 (1.65, 3.81) < 0.0001	2.06 (1.27, 3.34) 0.0034
**P for trend**	<0.0001	<0.0001	0.0014

Model 1: we did not adjust other covariates

Model 2: we adjusted age, sex, smoking status,alcoholic consumption, exercise habits, BMI,and SBP.

Model 3: we adjusted age, sex, smoking status,alcoholic intake, exercise habits, BMI,SBP, ALT; AST, GGT,HDL-C,TC

### Sensitive analysis

To evaluate the robustness of the primary findings, we performed multiple sensitivity analyses (Table 4). By restricting the sample to non-obese individuals defined as body mass index < 30 kg/m²(Model 4), the association between the TyH-i and diabetes remained unchanged (HR: 1.78; 95% CI: 1.26-2.49; P = 0.0009). A similar situation was also observed in non-drinkers (Model 5, HR: 1.93; 95% CI: 1.02-3.64; P = 0.0440), highlighting the consistency of this association in clinically relevant subgroups.

**Table 4:** The association between TyH-i and the onset of diabetes in different sensitivity analyses.

Variable	Model 4 HR 95%CI P	Model 5 HR 95% CI P
TyH-i	1.78 (1.26, 2.49) 0.0009	1.93 (1.02, 3.64) 0.0440
TyH-i (quartile)		
Q1	Ref	Ref
Q2	1.01 (0.61, 1.67) 0.9807	0.57 (0.23, 1.44) 0.2374
Q3	1.23 (0.74, 2.04) 0.4341	1.04 (0.44, 2.45) 0.9216
Q4	1.86 (1.12, 3.11) 0.0168	1.83 (0.76, 4.39) 0.1768
P for trend	0.0040	0.0546

Model 4 was sensitivity analysis in participants with BMI < 30 kg/m^2^. We adjusted for sex, age, BMI, alcoholic intake, smoking status, exercise habits, SBP, ALT, AST, GGT, TC and HDL-C.

Model 5 was sensitivity analysis in individuals without alcoholic intake. We adjusted for sex, age, BMI, alcoholic intake, smoking status, exercise habits, SBP, ALT, AST, GGT, TC and HDL-C.

HR: hazard ratio; CI: confidence interval; Ref: Reference; TyH-i: triglyceride-glycated hemoglobin index.

### The non-linear association between TyH-i and diabetes

**Fig 3:**
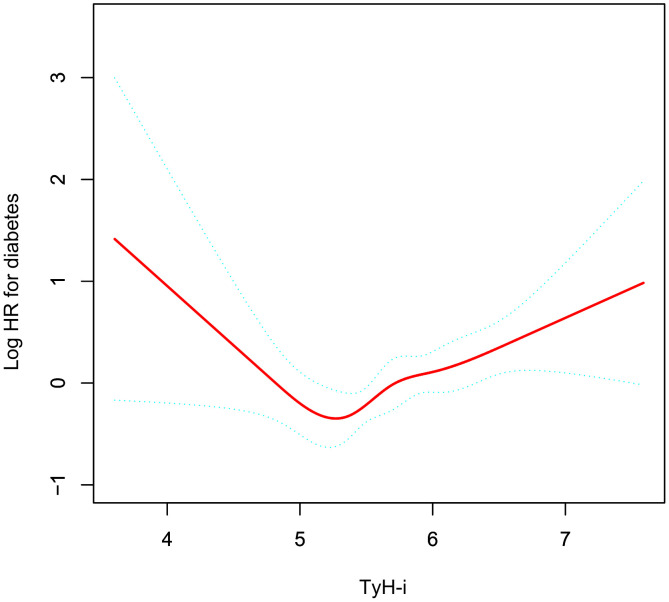
The non-linear relationship between TyH-i and the risk of T2D.

As shown in Table 5 and Fig 3, the association between the TyH-i and incident diabetes is non-linear. There is a turning point at 4.95. Below this threshold, higher TyH-i is linked to a lower diabetes risk (HR = 0.20; 95% CI: 0.05-0.78; P = 0.0206). Once this index exceeds 4.95, the increase in TyH-i also increases the risk of diabetes.For every additional unit increase, the risk of diabetes rises sharply and significantly (HR = 2.01; 95% CI: 1.45-2.79; P < 0.0001).

**Table 5:** The result of the two-piecewise Cox proportional hazards regression model.

Incident Diabetes	HR 95%CI	P
Fitting model by two-piecewise Cox proportional hazards regression		
**The inflection point of TyH-i**	4.95	
**≤4.95**	0.20 (0.05, 0.78)	0.0206
**>4.95**	2.01 (1.45, 2.79)	<0.0001
**P for the log-likelihood ratio test**	0.008	

We adjusted sex, age, BMI, alcoholic intake, smoking status, exercise habits, SBP, ALT, AST, GGT, TC and HDL-C.

HR: hazard ratios; CI: confidence; TyH-i: triglyceride-glycated hemoglobin index

### Subgroup analysis

The subgroup findings are summarized in Table 6. These analyses were designed to identify potential modifying factors that might affect the relationship between TyH-i and T2D. Prespecified and exploratory stratifications by sex, BMI, total alcohol consumption, age, smoking, habit of exercise, and BMI revealed that no significant interaction terms were uniformly non-significant (all P interaction > 0.05).

**Table 6:** Effect size of TyH-i on diabetes in prespecified and exploratory subgroups.

Characteristic	No of patients	HR 95%CI	P	P for interaction
Age(years)				0.8792
<45	1431	1.64 (1.06, 2.54)	0.0278	
45-60	1192	1.79 (1.11, 2.89)	0.0163	
≥60	118	2.37 (0.54, 10.40)	0.2510	
Sex				0.6351
Female	486	2.03 (1.03, 3.98)	0.0407	
Male	2285	1.71 (1.23, 2.39)	0.0015	
Total alcohol consumption (g/wk)				0.2965
=0	746	1.93 (1.09, 3.41)	0.0243	
>0	1995	1.66 (1.17, 2.35)	0.0047	
Smoking status				0.5495
Never-smoker	1226	2.06 (1.19, 3.58)	0.0103	
Past-smoker	726	2.04 (1.14, 3.65)	0.0169	
Current-smoker	789	1.44 (0.88, 2.36)	0.1470	
Exercise habits				0.9593
No	2340	1.77 (1.27, 2.46)	0.0008	
Yes	401	1.72 (0.61, 4.83)	0.3047	
BMI (kg/m^2^)				0.8086
<25	1337	1.90 (1.13, 3.21)	0.0162	
25-30	1185	1.67 (1.07, 2.62)	0.0239	
≥30	219	1.36 (0.57, 3.26)	0.4853	

Note 1: The above model adjusted for we adjusted for sex, age, BMI, alcoholic intake, smoking status, exercise habits, SBP, ALT, AST, GGT, TC and HDL-C.

Note 2: The model is not adjusted for the stratification variable in each case.

### Diagnostic performance of TyH-i in identifying diabetes

**Fig 4:**
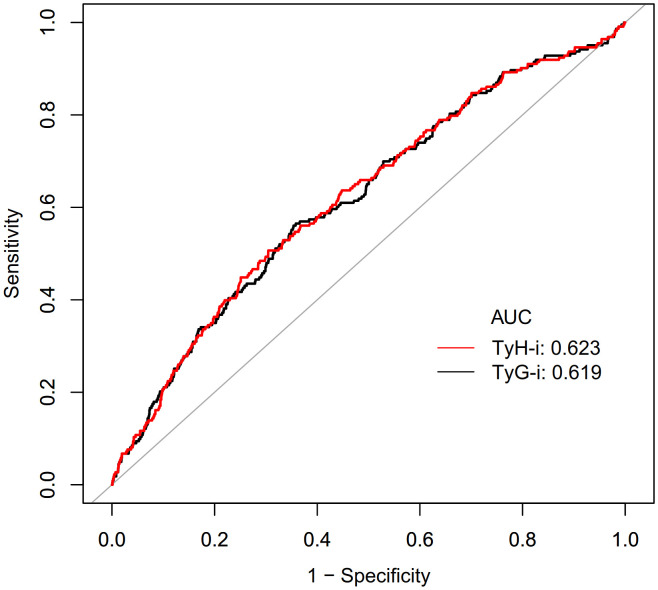
Diagnostic performance of TyH-i and TyG-i in identifying diabetes.

The diagnostic performance of TyH-i for identifying diabetes was examined using receiver operating characteristic analysis (Table 7, Fig 4). The area under the ROC curve was 0.623 (95% CI 0.5837-0.6617), indicating that it has a certain degree of discrimination ability. The optimal cut-off point for TyH-i was determined to be 5.9342, with a corresponding specificity of 69.54% and sensitivity of 50.67%. The resulting Youden index was 0.2021. The AUC of TyG-i was 0.619 (95% CI: 0.5804-0.6585). The optimal cut point for TyG-i was identified as 8.7750, yielding a specificity of 64.18% and a sensitivity of 56.50%.Consequently, the Youden index was calculated to be 0.2068. The Youden values produced by the TyG-i and the TyH-i are almost the same, and the research results indicate that the AUC of TyH-i is slightly higher than that of TyG-i, suggesting that it has the potential to serve as a reliable marker for identifying diabetes.

**Table 7:** The area under the receiver operating characteristic curve for each assessment parameter in identifying diabetes.

Test	AUC	95%CI	Best threshold	Specificity	Sensitivity	Youden Index
TyH-i	0.623	0.5837-0.6617	5.9342	0.6954	0.5067	0.2021
TyG-i	0.619	0.5804-0.6585	8.7750	0.6418	0.5650	0.2068

TyH-i: triglyceride-glycated hemoglobin index; TyG-i: triglyceride-glucose index

## Discussion

In a cohort of 2,741 individuals with NAFLD, higher TyH-i concentrations were associated with an increased likelihood of developing type 2 diabetes (T2D). Furthermore, the analysis identified a U-shaped association between TyH-i and T2D risk, indicating elevated risk at both lower and higher ranges of TyH-i. TyH-i and TyG-i demonstrated similar discriminatory capabilities. Furthermore, both the sensitivity analysis and the subgroup analysis confirmed the above results, further strengthening the robustness of our conclusion.

Impaired insulin function is the main defect in the development of diabetes. It coordinates the continuous process from the pre-diabetic stage to obvious hyperglycemia [20]. As two determinants of the TyH index, fasting triglycerides and glycated hemoglobin are both closely related to the disorder of insulin action and the broader metabolic balance [21–24]. Extensive prior research has clarified the interplay among triglycerides, glycated hemoglobin, and diabetes onset. A large retrospective cohort that followed more than 30,000 individuals, for example, used multivariable modelling to show that even triglyceride concentrations remaining within the conventionally accepted reference interval were remained an independent predictor of heightened diabetes risk over follow-up [25,26]. A comprehensive meta-analysis of 16 prospective studies (n = 44,203) further confirmed that glycated hemoglobin is a powerful and independent predictor of new-onset diabetes. Relative to participants with glycated hemoglobin levels below the prediabetic threshold, individuals with HbA1c values in the 5.7%−6.4% range exhibited a markedly increased risk of progressing to overt diabetes [27]. Overall, these data highlight the crucial role of circulating triglycerides and glycated hemoglobin in metabolic disorders, as well as their value in predicting the onset of diabetes. A study has shown that TyH-i is independently associated with an increased risk of type 2 diabetes [17]. Any increase in this comprehensive index necessarily implies an increase in fasting triglycerides or HbA1c (or both) – thereby indicating that the glucose-lipid balance is deteriorating. NAFLD represents a prevalent chronic hepatic disorder whose pathophysiology is intimately intertwined with the cluster of abnormalities that define metabolic syndrome [28]. Prior research indicates that individuals with NAFLD experience a markedly elevated incidence of diabetes [29]. However, in the NAFLD population, evidence linking the triglyceride-glycated hemoglobin index to incident T2D remains scarce. In the present analysis, TyH-i values exceeding 4.95 were associated with a higher risk of diabetes among participants with NAFLD. These findings suggest that risk stratification and timely intervention guided by TyH-i may help mitigate the likelihood of diabetes in this high-risk group.

After adjusting for potential confounders, a U-shaped association emerged between TyH-i and the risk of T2D. Specifically, when TyH-i was below 4.95, each one-unit increase corresponded to an approximate 75% reduction in T2D risk. In contrast, once TyH-i exceeded 4.95, higher values were linked to an elevated likelihood of T2D. Our analysis revealed a U-shaped relationship between TyH-i and the risk of developing diabetes. While the increasing risk at higher TyH-i levels is robustly supported by a substantial number of events, the descending arm of this U-shape, representing lower TyH-i values, should be interpreted with caution. The limited number of diabetes events in this segment (n = 12) may have influenced the stability and precision of the observed association. Therefore, while our piecewise model identified a non-linear trend, further research with larger sample sizes encompassing a wider range of lower TyH-i values is needed to definitively confirm the protective effect observed below the inflection point.

While higher TyH-i is positively correlated with T2D risk in line with established metabolic theories, the inverse association at TyH-i below 4.95 needs cautious interpretation. This unexplained paradoxical finding may stem from population-specific characteristics in this Japanese NAFLD cohort. Excessively low triglycerides may indicate advanced hepatic impairment, malnutrition or frailty, thereby altering metabolic status. Moreover, residual confounding from unmeasured factors such as genetics, extreme diets and concomitant medications may affect individuals with low TyH-i. The clinical relevance of this inverse trend remains unclear, and further prospective studies with detailed clinical indicators are necessary to elucidate the U-shaped relationship. Persistently elevated triglyceride levels promotes hepatic steatosis, which, in turn, drives hepatic triglyceride overproduction and progressively impairs insulin sensitivity [30]. This dysmetabolic state accelerates lipogenesis, further undermines insulin’s capacity to control glucose homeostasis, and intensifies hepatic lipid deposition, ultimately culminating in impairment of pancreatic beta-cell function [31]. The accumulation of lipid droplets in pancreatic islets can interfere with glucose-induced insulin secretion, thereby promoting the occurrence of diabetes [32,33]. Moreover, lower TyH-i values have also been linked to a heightened likelihood of diabetes. It is worth noting that, despite having higher levels of insulin resistance or diabetes risk factors, the triglyceride levels in the African population were unexpectedly low. This phenomenon may be attributed to the fact that hyperinsulinemia inhibits the activity of insulin-sensitive lipase, thereby reducing the release of free fatty acids from adipose tissue [34–36]. Furthermore, Carriers of the PNPLA3 I148M variant exhibit decreased circulating triglycerides, but also show exacerbated insulin resistance and increased risk of diabetes [37]. Pancreatic α-cells play a important role in sustaining metabolic homeostasis by coordinating the regulation of glucose, amino acid, and lipid pathways [38]. Abnormal function of these cells can lead to hypoglycemia, suggesting that the regulation of α-cells is disrupted, which is a core pathogenic link in the occurrence and development of diabetes [39].

Although the NAGALA cohort has been widely used, this secondary analysis still holds unique academic value. Unlike previous studies that mostly focused on the association of a single indicator and had relatively simple analytical dimensions, this study has achieved innovation in multiple aspects, such as multi-factor interaction, subgroup stratification, more rigorous statistical strategies, and comprehensive adjustment for confounding factors. It not only addresses the shortcomings of previous research but also provides more reliable evidence for clinical practice. Conducting in-depth mining based on public data not only saves research resources but also extends and breaks through existing achievements, providing new ideas for related fields. This investigation offers several notable strengths. By leveraging a large cohort and a prolonged follow-up, it provides a robust and dependable evaluation of the relationship between TyH-i and incident diabetes. First, we identified a U-shaped pattern that turning point the inflection threshold at which TyH-i modifies the risk of type 2 diabetes. Second, the application of multiple statistical frameworks, supplemented by extensive sensitivity tests, strengthens the credibility of the findings and supports their generalizability. Third, by making a parallel comparison between TyH-i and TyG-i, we have provided an important basis for evaluating the relative efficacy of the two in predicting diabetes.

However, our investigation still has many shortcomings. First of all, although we accounted for a series of potential confounding factors, we still cannot completely eliminate the influence of residual confounding. Secondly, the research population was solely from Japan, which may limit the generalizability of the research results to other ethnic groups. Thirdly, because this work used a retrospective cohort design, establishing causality between TyH-i and diabetes risk remains beyond its scope; confirmation will require controlled experiments or intervention studies. Finally, in the absence of serial TyH-i assessments, it is impossible to assess the impact of the dynamic changes of this indicator over time on the risk of type 2 diabetes. Future studies should strengthen the dynamic monitoring of confounding variables, especially the fluctuations of HbA1c and TG during the follow-up period, in order to more clearly elucidate the impact of TyH-i changes on the subsequent risk of T2D.

## Conclusion

This study demonstrates a U-shaped association between TyH-i and the risk of type 2 diabetes among adults with NAFLD. These findings suggest that timely TyH-i guided interventions may help lower the likelihood of developing type 2 diabetes in this population.

## Supporting information

S1 Table The results of the collinearity screening. Collinearity diagnostics revealed that diastolic blood pressure (DBP) was highly correlated with other predictors, and thus was excluded from the final model.
(DOCX)

S2 Table The relationship between HbA1c,TG and the onset of diabetes under different models. S2 Table shows the relationship between TG and HbA1C as independent predictors and the incidence of diabetes.
(DOCX)

## Abbreviations

T2D: type 2 diabetes
NAFLD: Non-alcoholic fatty liver disease
TyG-i: triglyceride-glucose index
HbA1c: glycated hemoglobin
TyH-i: triglyceride-glycated hemoglobin index
FPG: fasting plasma glucose
BMI: body mass index
TG: triglyceride
TC: total cholesterol
ALT: alanine aminotransferase
AST: aspartate aminotransferase
HDL-C: high-density lipoprotein cholesterol
GGT: gamma-glutamyl transferase
DBP: diastolic blood pressure
SBP: systolic blood pressure
Scr: serum creatinine
ROC: receiver-operating characteristic
AUC: area under the curve
SD: standard deviations
HR: hazard ratio
CI: confidence interval
