# Supplementary material for: Association between the triglyceride-glycated hemoglobin index and diabetes risk among patients with Non-alcoholic fatty liver disease: A longitudinal cohort study
Source: PLoS One. 2026 Jun 5;21(6):e0350633. doi: 10.1371/journal.pone.0350633 (PMC13240927; doi:10.1371/journal.pone.0350633)
Supplement: S1 Table — Collinearity diagnostics revealed that diastolic blood pressure (DBP) was highly correlated with other predictors, and thus was excluded from the final model. (DOCX) [file pone.0350633.s001.docx]

**S1 Table. The results of the collinearity screening**

|  | Step 1 | Step 2 |
| --- | --- | --- |
| Gender | 1.5 | 1.5 |
| Age(years) | 1.2 | 1.2 |
| Drinking status | 1.2 | 1.2 |
| Smoking status | 1.2 | 1.2 |
| Habit of exercise | 1 | 1 |
| SBP (mmHg) | 5.6 | 1.2 |
| DBP (mmHg) | 5.7 | NA |
| BMI (kg/m^2^) | 1.3 | 1.3 |
| ALT (U/L) | 3.6 | 3.6 |
| AST (U/L) | 3.2 | 3.2 |
| HDL-C (mmol/L) | 1.3 | 1.3 |
| GGT (U/L) | 1.4 | 1.4 |
| TC (mmol/L) | 1.1 | 1.1 |
| FPG(mmol/L) | 1.1 | 1.1 |
