# Supplementary material for: Association between the triglyceride-glycated hemoglobin index and diabetes risk among patients with Non-alcoholic fatty liver disease: A longitudinal cohort study
Source: PLoS One. 2026 Jun 5;21(6):e0350633. doi: 10.1371/journal.pone.0350633 (PMC13240927; doi:10.1371/journal.pone.0350633)
Supplement: S2 Table — shows the relationship between TG and HbA1C as independent predictors and the incidence of diabetes. (DOCX) [file pone.0350633.s002.docx]

**S2 Table The relationship between HbA1c,TG and the onset of diabetes under different models**

| **Variable** | **Model 1**  **HR 95%CI P** | **Model 2**  **HR 95%CI P** | **Model 3**  **HR 95%CI P** |
| --- | --- | --- | --- |
| **diabetes** |  |  |  |
| HbA1c | 21.91 (14.26, 33.64) <0.0001 | 19.34 (12.37, 30.23) <0.0001 | 19.20 (12.16, 30.34) <0.0001 |
| TG | 1.00 (1.00, 1.00) <0.0001 | 1.00 (1.00, 1.01) <0.0001 | 1.00 (1.00, 1.00) 0.0080 |

Model 1: we did not adjust other covariates

Model 2: we adjusted age, sex, smoking status,alcoholic consumption, exercise habits, BMI,and SBP.

Model 3: we adjusted age, sex, smoking status,alcoholic intake, exercise habits, BMI,SBP, ALT; AST, GGT,HDL-C,TC
